# Retraction: Fishing grounds footprint and economic freedom indexes: Evidence from Asia-Pacific

**DOI:** 10.1371/journal.pone.0346950

**Published:** 2026-04-13

**Authors:** The PLOS One Editors

The *PLOS One* Editors retract this article [1] due to concerns about:

- Non-compliance with PLOS policies on Authorship.
- High similarity with a study and results previously reported in [2] that were not cited or discussed in [1].
- The use of terminology that differs from standards in the field (e.g., “monetary development” instead of “economic development”, “get right of entry to goods and aids” instead of “access goods and services”).

SA did not agree with the retraction. CL, YAK, and AB either did not respond directly or could not be reached.

## References

[1] Amin S, Li C, Khan YA, Bibi A. RETRACTED: Fishing grounds footprint and economic freedom indexes: Evidence from Asia-Pacific. PLoS One. 2022;17(4):e0263872. doi: 10.1371/journal.pone.0263872 35417457 PMC9007340

[2] Karimi MS, Khezri M, Khan YA, Razzaghi S. Exploring the influence of economic freedom index on fishing grounds footprint in environmental Kuznets curve framework through spatial econometrics technique: evidence from Asia-Pacific countries. Environ Sci Pollut Res Int. 2022;29(4):6251–66. doi: 10.1007/s11356-021-16110-8 34453249

